# Cor Triatriatum: an uncommon congenital anomaly - the experience of a tertiary care center in a developing country

**DOI:** 10.3389/fcvm.2025.1531754

**Published:** 2025-03-04

**Authors:** Jad Abdul Khalek, Christophe El Rassi, Maria Abou Mansour, Bshara Sleem, Issam El Rassi, Fadi Bitar, Mariam Arabi

**Affiliations:** ^1^ Faculty of Medicine, American University of Beirut Medical Center, Beirut, Lebanon; ^2^ Department of Pediatric Cardiac Surgery, Al Jalila Children’s Specialty Hospital, Dubai, United Arab Emirates; ^3^ Division of Pediatric Cardiology, Department of Pediatric and Adolescent Medicine, American University of Beirut Medical Center, Beirut, Lebanon

**Keywords:** Cor Triatriatum, echocardiography, congenital heart surgery, congenital heart disease, blood flow obstruction

## Abstract

**Background:**

Cor Triatriatum is a congenital anomaly characterized by the abnormal presence of a fibromuscular junction in one of the atria, as seen on echocardiography. This anomaly can lead to major hemodynamic problems and obstruction of blood flow. This study aims to explore the risk factors, diagnostic modalities, and surgical interventions used to tackle this congenital anomaly at a tertiary care center over an 18-year period.

**Materials and methods:**

Medical records of congenital heart disease patients at the Children's Heart Center at the American University of Beirut Medical Center between 2006 and 2024 were retrospectively reviewed. Data collection included demographic characteristics, clinical outcomes, hospitalization details, and surgical treatment. Ethical approval was obtained, and descriptive statistics were employed for data analysis using SAS 9.4.

**Results:**

At our center, 7 patients were diagnosed with Cor Triatriatum, with a median age of 5 months. 4 of the patients were female, 3 were males, and the median hospital stay was 7 days. All patients were diagnosed with Cor Triatriatum Sinister, and respiratory symptoms were prevalent. Pulmonary vein abnormalities were observed in 4 ouf of 7 (57.1%) patients and atrial septal defects in 2 out of 7 patients (28.5%). Surgery resulted in successful membrane resection for all operated patients, with significant symptom improvement postoperatively.

**Conclusion:**

Cor Triatriatum is a rare congenital anomaly requiring early detection and diagnosis. Surgical intervention remains the mainstay of treatment, with favorable outcomes when performed promptly. Larger studies are recommended to optimize management strategies and improve long-term outcomes for affected patients.

## 1 Introduction

Congenital heart disease is a significant category of congenital problems that is the leading cause of death in infants due to birth defects (1, 2). The treatment and management of congenital heart disease have seen considerable progress over the years (3). John Gibbon achieved a significant milestone in congenital heart disease therapy with the first use of a cardiopulmonary bypass in 195 (4). Advancements in diagnostic methods, especially echocardiography, greatly enhanced our capacity to identify abnormalities over time (5). Nevertheless, complex congenital heart disease continues to present abundant diagnostic and therapeutic challenges.

**Figure 1:**
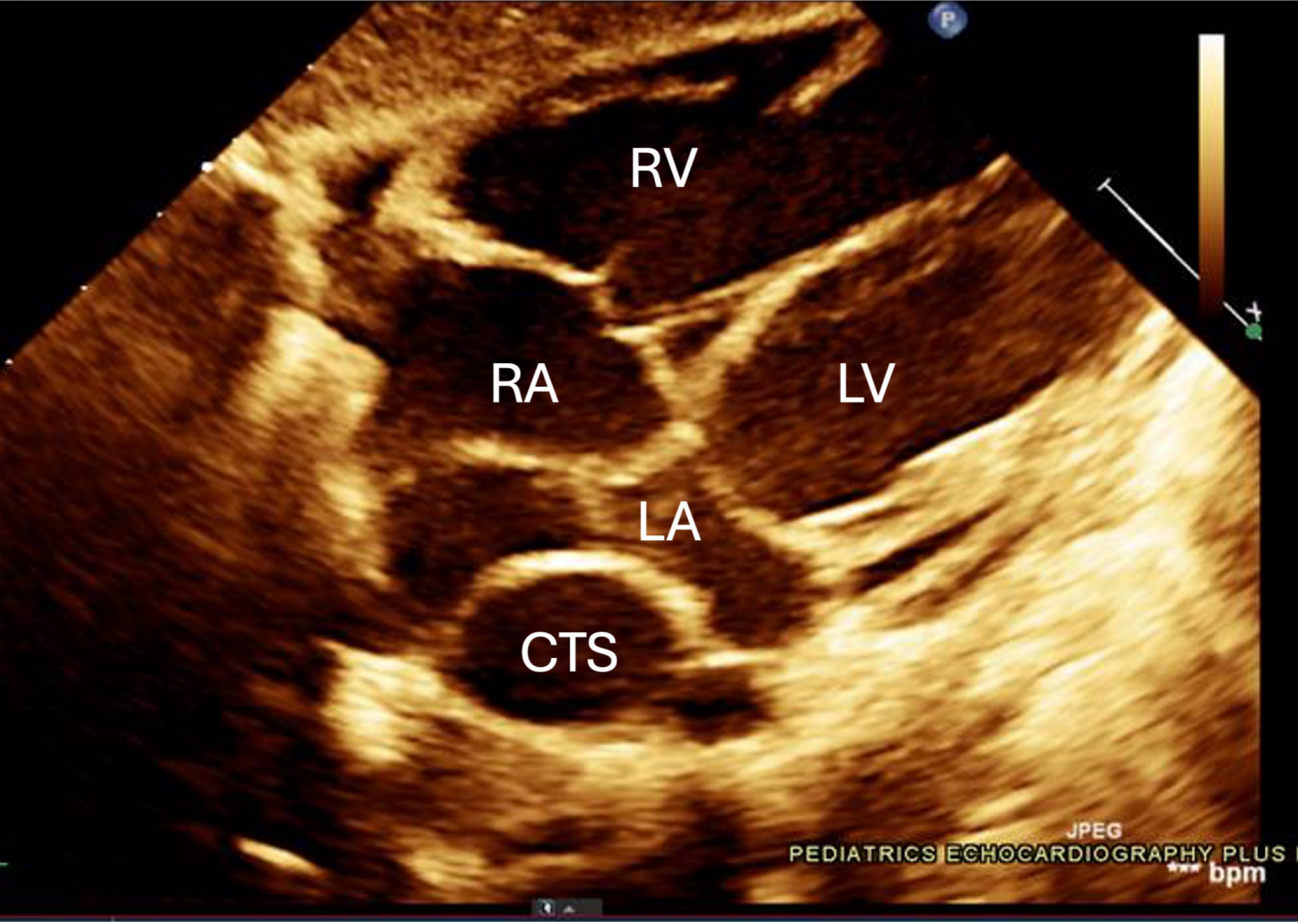
The echocardiogram shows an abnormal membrane or fibromuscular partition that divides the left atrium into two chambers. This partition separates the pulmonary venous inflow, creating a proximal and distal chamber. Notable in this image are the membranous structures and potential flow obstruction, which are typical in Cor Triatriatum cases. The relevant chambers are labeled with *RA: Right Atrium, *RV: Right Ventricle, *LA: Left Atrium, *LV: Left Ventricle, *CTS: Cor Triatriatum Sinister.

**Figure 2:**
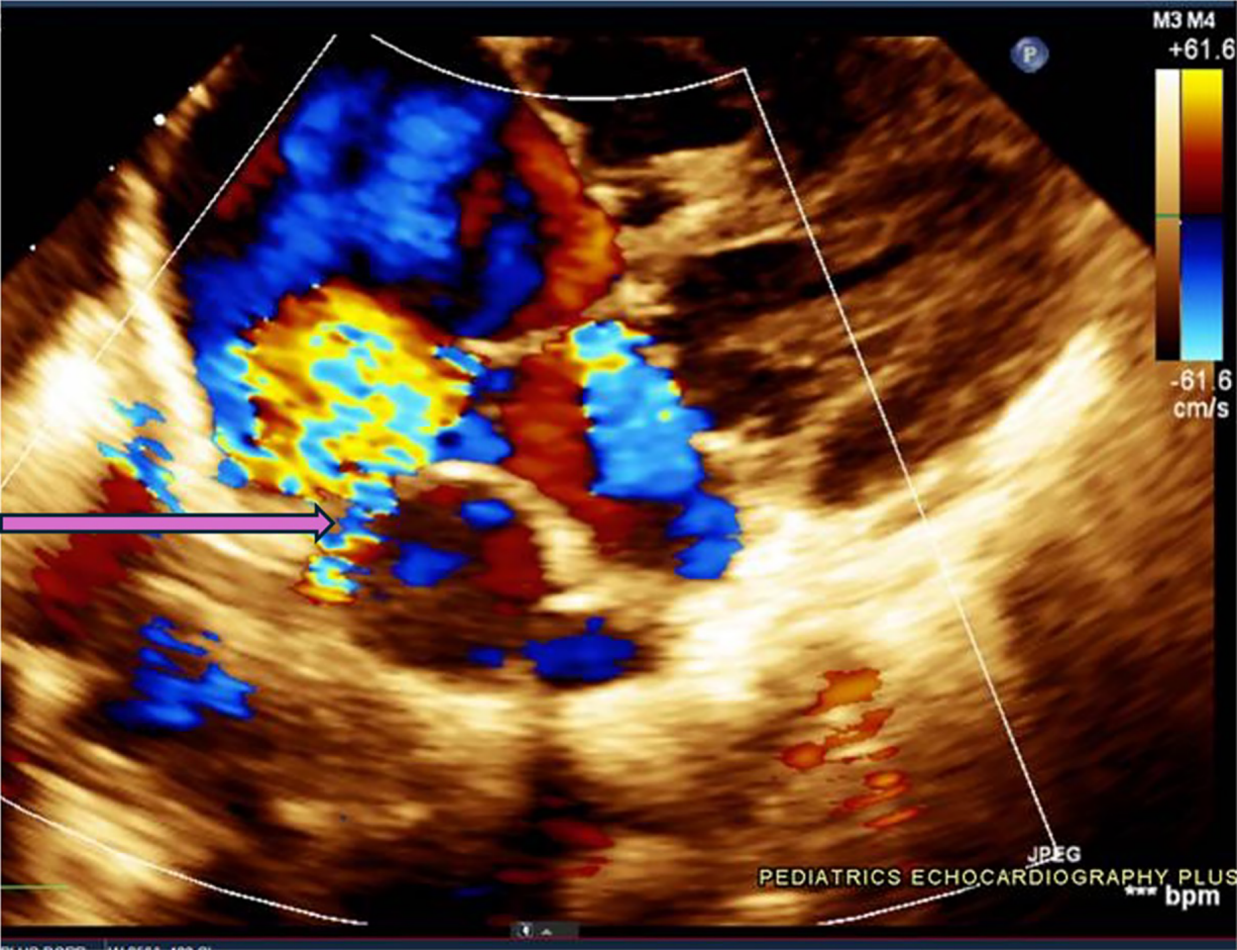
This echocardiogram highlights the turbulent flow pattern within the left atrium, as seen in the color Doppler display. The blue and red color signals indicate a high-velocity flow across the membranous partition within the atrium, characteristic of Cor Triatriatum. The arrow is pointing towards a small restrictive communication present in the membrane, reinforcing the diagnosis and illustrating potential hemodynamic implications.

**Figure 3:**
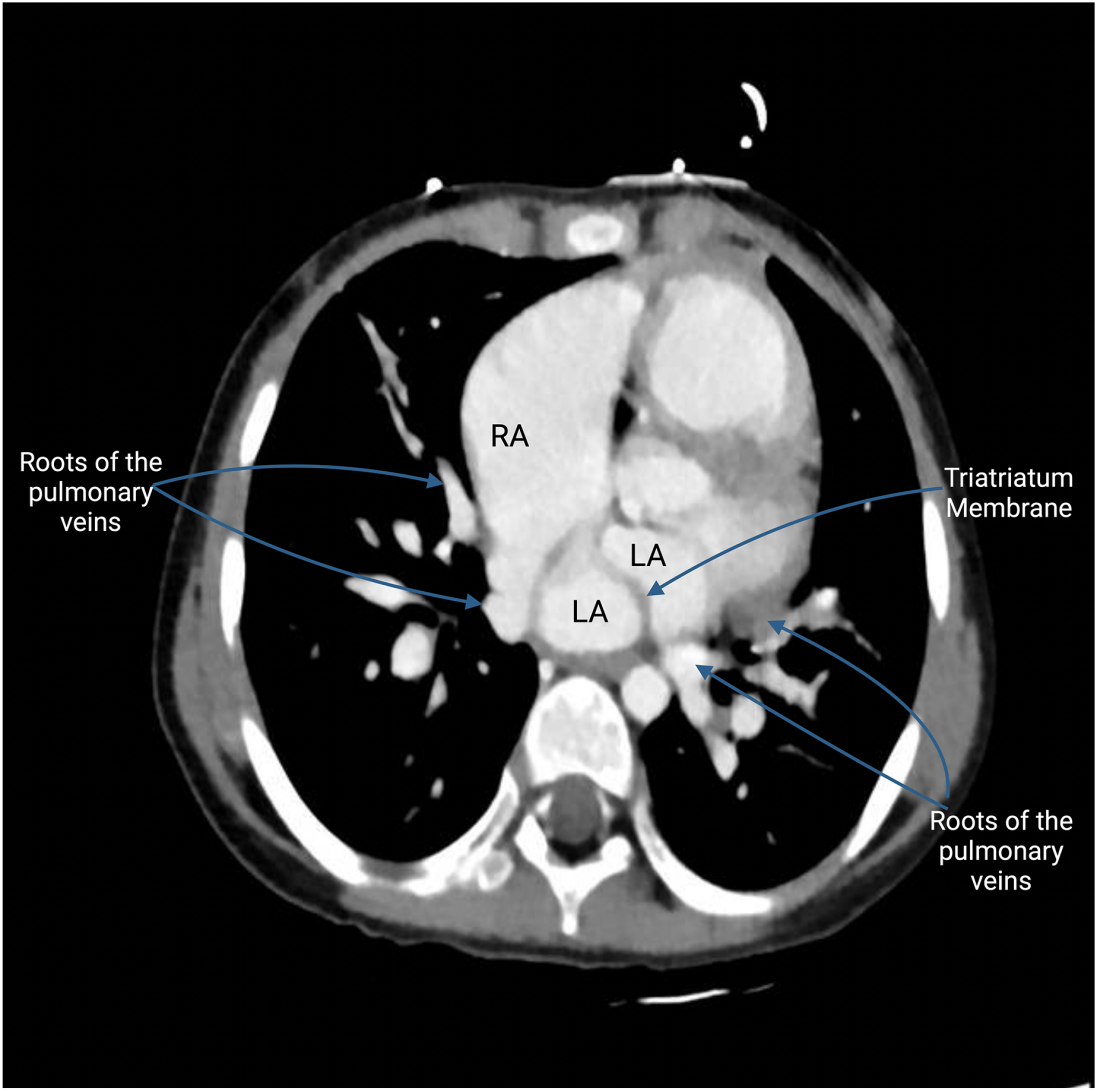
This CT image highlights the fibromuscular membrane dividing the left atrium into two chambers. Visible on the scan is the Cor membrane, which acts as a partition within the atrium, creating a distinct proximal and distal chamber. The proximal chamber receives the pulmonary veins, while the distal chamber channels blood to the mitral valve, leading to the left ventricle. The scan shows the unusual entry points of the pulmonary veins: the roots of two pulmonary veins are depicted entering the roof of the right atrium, an anomalous connection indicative of PAPVR. In contrast, the roots of the two other pulmonary veins are seen entering the left atrium, following the normal anatomical pattern. This difference between normal and abnormal venous connections highlights the unique challenges posed by Cor Triatriatum.

One such congenital anomaly is Cor Triatriatum, a condition characterized by the presence of an abnormal fibromuscular septum inside one of the atria, dividing it into three chambers instead of the usual two (6). The name “Cor Triatriatum” was coined by Borst in 1905 to describe a septum in the left atrium (7). Figure 1 represents a transthoracic echocardiogram showing the prevalent anatomy of Cor Triatriatum. This anomaly can occur in the left atrium (Cor Triatriatum Sinister) or, less commonly, in the right atrium (Cor Triatriatum Dexter), leading to major hemodynamic turbulence due to obstruction of blood flow (7). The two chambers are typically connected by a small opening, through which the membrane acts as an obstruction to pulmonary venous drainage, ultimately leading to the patient's symptoms. Figure 2 represents a color Doppler echocardiogram demonstrating the turbulent flow across the atrial membrane. Figure 3 highlights the obstruction caused by the triatriatum membrane in a Computed Tomography (CT) image with its associated pulmonary veins. Cor Triatriatum presents as a spectrum of clinical manifestations, from asymptomatic cases detected incidentally to severe cases presenting with heart failure, syncope, and sudden cardiac arrest (8).

Cor Triatriatum is classified according to various systems, such as the Loeffler classification (9), Lam classification (6), the modified Lucas classification (10), and the newly proposed Mashadi-Narasimhan-Said classification (11). Echocardiography is the diagnostic modality of choice for Cor Triatriatum (12, 13). While cardiac angiography and catheterization were widely used in the past, they are now rarely utilized and largely supplanted by echocardiography, but can still confirm the diagnosis by displaying differential atrial chamber filling (14).

The primary pathophysiological mechanism of Cor Triatriatum involves the obstruction of blood flow due to an abnormal septum in the atria, leading to increased atrial pressures and subsequent pulmonary and systemic complications (6, 15). Table 1 highlights the three prevailing theories on the formation of Cor Triatriatum (16–18). The definitive treatment for Cor Triatriatum involves surgical correction, typically performed under cardiopulmonary bypass (19). The first successful surgical correction of Cor Triatriatum was reported in 1956 by Lam et al., marking a significant milestone in the management of this anomaly (20). Since then, surgical excision of the abnormal septum under cardiopulmonary bypass has become the definitive treatment, with a very low risk of recurrence (10).

**Table 1:** Theories of embryonic formation of Cor Triatriatum.

Theory	Description	Consequences
Malseptation Theory	Abnormal membrane results from atypical growth of the septum primum.	Septum-like structure in the atrium leading to obstruction of pulmonary venous return, increased venous pressure, and secondary pulmonary hypertension.
Entrapment Theory	Common pulmonary vein becomes trapped in the embryonic sinus venosus, preventing its incorporation into the atrium.	
Malincorporation Theory	Failure in the normal incorporation of the pulmonary vein into the atrium during fetal development, leading to the formation of a fibromuscular membrane.	

Despite advancements in diagnostic and surgical techniques, Cor Triatriatum remains challenging due to its rarity and the potential for delayed diagnosis (21). This study aims to explore the prevalence, risk factors, and possible surgical interventions, as well as multimodal diagnostic modalities for better treatment in pediatric patients with Cor Triatriatum.

## 2 Methods

This study involved a retrospective chart review of medical records for pediatric patients diagnosed with Cor Triatriatum at the American University of Beirut Medical Center's Children's Heart Center from 2006 to 2024. 7 patients were identified with Cor Triatriatum. Due to the retrospective nature of the study, all patients were deidentified, stored securely, and accessible only to the research team. The study was approved by the Biomedical Institutional Review Board (BIO-2024-0160) and adhered to ethical standards as outlined in the Declaration of Helsinki, the Nuremberg Code, and the Belmont Report.

**Figure 4:**
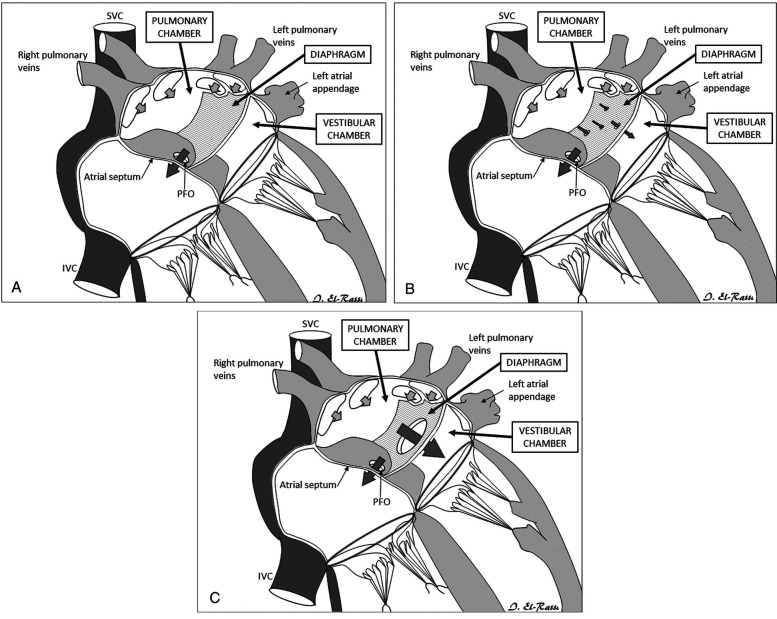
**(A)** Group 1 Loeffler classification of Cor Triatriatum. **(B)** Group 2 Loeffler classification of Cor Triatriatum. **(C)** Group 3 Loeffler classification of Cor Triatriatum.

Data were collected on patient demographics (age, gender, height, and weight), clinical presentations, and diagnostic imaging results, including echocardiography, chest x-rays, and electrocardiograms to assess the morphology of the anomaly and the extent of obstruction. Criteria for diagnosis included the visualization of a membrane dividing the left or right atrium into two distinct chambers with evidence of blood flow obstruction. Additionally, information on surgical interventions and postoperative outcomes, including complications and length of hospital stay was gathered. Loeffler's classification was used for Cor Triatriatum morphology (Figure 4). Data on surgical treatment were highlighted, including type of procedure, intraoperative findings, and immediate postoperative outcomes. Long-term follow-up was also documented highlighting long-term postoperative outcomes and the patient's status during office visits.

Descriptive statistics were used to showcase continuous variables, which were reported as medians with interquartile ranges based on their distribution. Categorical variables were mostly presented as frequencies. All data analysis was conducted using SAS 9.4 (SAS Institute, Cary, NC, USA).

## 3 Results

At our center, 7 cases of Cor Triatriatum were diagnosed. The median interquartile range (IQR) age of the identified 7 patients was 5 (4–23.0) months, with a median (IQR) body weight of 14 (9.8–14.0) kg and a median (IQR) height of 92 (80.0–92.75) cm. Of the 7 patients, 4 were females and 3 were males. Table 2 outlines the demographic characteristics of these patients.

**Table 2:** Demographic characteristics for patients with Cor Triatriatum.

Demographic characteristics	Patients with Cor Triatriatum (*N* = 7)
Age (months) median (IQR)	5 (4–23.0)
Females *n*	4
Males *n*	3
Weight (kg) median (IQR)	14 (9.8–14.0)
Height (cm) median (IQR)	92 (80.0–92.75)
Cor Triatriatum Sinister *n*	7

The median duration of hospital stay was 7.0 days (5.5–8.0 days) encompassing the time required for both the diagnosis and the surgical intervention of the patients. The cohort included. 4 patients were admitted to the pediatric intensive care unit, and the remaining 3 were managed as floor patients. No patients were admitted to the neonatal intensive care unit. Surgical intervention was performed in 6 out of 7 (85.7%) of the patients. The patients who underwent surgery had successful resections of the obstructing membrane, with significant improvement in clinical symptoms and stabilization of their conditions. Patient outcomes are reported in Table 3.

**Table 3:** Clinical outcomes and hospitalization details for patients with Cor Triatriatum.

Clinical outcomes	Patients with Cor Triatriatum (*N* = 7)
Median length of hospital stay (days) (IQR)	7.0 (5.5–8.0)
Hospital stay: Floor *n*	3
Hospital stay: Pediatric intensive care unit *n*	4
Hospital stay: Neonatal intensive care unit *n*	0
Underwent surgery *n*	6
Mortality *n*	0

Laboratory results demonstrated a median white blood cell count of 13,100/cu.mm, indicating a systemic inflammatory response. Furthermore, c-reactive protein levels were elevated, with a median value of 21.4 mg/L, further supporting the presence of an inflammatory state. The hemoglobin, hematocrit, mean corpuscular volume, mean corpuscular hemoglobin, median prothrombin time, partial thromboplastin time, and platelet counts were within physiological ranges.

All patients were diagnosed with Cor Triatriatum Sinister (CTS) and the diagnosis was made by echocardiography in all cases. The clinical presentation of patients with Cor Triatriatum was dominated by respiratory symptoms. Dyspnea was a common finding, reported in five patients, with one of these cases also showing signs of pulmonary hypertension. One patient presented with fast and labored breathing. Additionally, one patient had symptoms of an upper respiratory tract infection for two weeks prior, with minimal cough on presentation. Another patient exhibited both dyspnea and congestion. These findings highlight the predominantly respiratory nature of clinical symptoms in patients with Cor Triatriatum.

Atrial septal defects were present in 2 patients. Among the 7 patients with Cor Triatriatum, multiple pulmonary vein abnormalities were documented. One patient exhibited pulmonary veins draining into a chamber posterior to the left auricle, with a connection to the left auricle complicated by obstruction. Another patient showed obstructed venous flow due to the Cor Triatriatum septum itself. Pulmonary veins draining into a distal chamber were observed in one case, while another displayed right pulmonary veins with anomalous drainage into the right atrium, consistent with partial anomalous pulmonary venous return (PAPVR). The finding of PAPVR was also evident in another patient. The remaining 2 patients did not exhibit any associated pulmonary vein abnormalities. Among the 7 patients with Cor Triatriatum, various valvular abnormalities were documented. One patient had both aortic and mitral regurgitation, while another presented with mitral regurgitation alone, with the remaining 5 having normal valves. Radiographic evidence of pulmonary edema was observed in 5 patients.

Table 4 provides a comprehensive overview of each patient's associated anomalies, clinical presentations, atrial communication, and pulmonary vein anomalies, along with hospital stays and outcomes.

**Table 4:** Patient conditions and outcomes.

Patient#	Associated anomalies	Clinical presentation	Atrial communication	Anomalous pulmonary veins	Hospital stay	Outcome
Patient 1	Patent foramen ovale, small, connecting the vena cava with right auricle	Dyspneic	Left sided communication	Pulmonary veins draining into a chamber behind left auricle and that connects the left auricle with obstruction	7 days	Excellent result and no residual abnormalities. Patient was discharged home in good clinical condition.
Patient 2	Aortic and Mitral Regurgitation	Dyspneic	Left sided communication	Obstructed flow due to CTS	10 days	Good cardiac function post resection of membrane good flow to the pulmonary veins.
Patient 3	N/A	Fast and labored breathing	Left sided communication	N/A	5 days	Good results, feeding well. Normal S1 and S2 recorded, sinus rhythm, and no pathological murmur. Slight pericardial effusion seen.
Patient 4	Atrial Septal Defect	Dyspneic	Left sided communication	Pulmonary veins draining in a distal chamber	3 days	Advised to undergo resection of membrane but lost to follow up.
Patient 5	Atrial Septal Defect and Ellis-van Creveld syndrome	Upper respiratory tract infection since 2 weeks and minimal cough	Left sided communication	Right pulmonary veins anomalous drainage into the right atria and Partial anomalous pulmonary venous return	6 days	Cough resolved post-surgery and comfortable sleep noted. Clean surgical wound with no erythema. Surgery went well with no effusion.
Patient 6	Coxsackie virus infection and Mitral Regurgitation	Dyspneic	Left sided communication	Partial anomalous pulmonary venous return	8 days	Good outcome. No medications needed, and growth is normal. An oxygen saturation of 100% is noted.
Patient 7	Viral respiratory infection	Dyspneic and congested	Left sided communication	N/A	8 days	No medications. Oxygen saturation is 100%. Electrocardiogram and echocardiography are normal

6 patients underwent operations for resection of the membrane. Surgical interventions varied across the 6 patients with Cor Triatriatum, each addressing unique structural abnormalities. Cardiopulmonary bypass with aortic cross-clamping was consistently utilized. The cross-clamp time required did not exceed half an hour and the bypass time did not take more than one hour for each individual patient. This step was followed by atriotomies and targeted membrane resections. In three cases, specific approaches were taken to either redirect pulmonary veins by resecting the membrane or reinforcing the mitral valve through mitral valve repair surgery in cases of severe mitral regurgitation. Additionally, some procedures involved the use of autologous pericardial patches for atrial septal repairs and to guide pulmonary venous flow. Each surgery concluded with chest drainage and sternotomy closure, highlighting the individualized yet systematic approach to managing these complex cardiac anomalies. One patient was lost to follow-up.

## 4 Discussion

Cor Triatriatum is a rare congenital disorder comprising 0.1%–0.4% of all congenital heart disorders (15). In particular, CTS presents with a septated left atrium, isolating a proximal part of the atrium with the pulmonary veins' entry from a distal part communicating with the left ventricle through the mitral valve (20, 22). With multiple theories discussed in the literature, the most accepted seems to be an abnormal incorporation of the common pulmonary vein in the left atrium (13, 15). Obstructive symptoms mimicking mitral stenosis and pulmonary vein stenosis may vary greatly depending on the communication level within the now two-chambered left atrium (6, 20, 22, 23). In fact, the Loeffler classification defines three possible presentations of CTS according to the amount of communication provided by the occluding diaphragm (9). A non-communicating diaphragm constitutes group 1 (Figure 4A). The septating membrane presents one or more small openings in group 2 (Figure 4B). Group 3 patients present with a large opening within the diaphragm (Figure 4C) (9, 13, 24). Therefore, a diagnosed case of asymptomatic CTS early in life, which is usually the case in a patient belonging to group 3 CTS, may delay diagnosis till a later age (13, 22). When diagnosed with CTS, regardless of age, patients are sent for surgical resection of the membrane occluding the left atrium, which is a curative management option (13, 25).

In this study, we have compiled data from a cohort of 7 patients, with a median age of 5 months, emphasizing the early presentation typically associated with this anomaly.

The median age at diagnosis for CTS is typically under 1 year, reflecting the early onset of symptoms associated with this congenital abnormality (26). In a Canadian study conducted at a tertiary care center involving 82 patients with CTS, the median age at presentation was 8 months (27). Similarly, an Australian study reported a median age of presentation of 6 months (28). These findings are consistent with those observed in our study. This can be attributed to the fact that the clinical manifestations of CTS, such as respiratory distress and signs of heart failure, often present early in life (26). Furthermore, early diagnosis of CTS is crucial for timely intervention, which may explain the comparable median ages at presentation across various studies (26–29). The physiological impact of CTS largely depends on the size of the opening between the accessory and the main atrial chambers (12). In infants and young children, this restricted flow leads to pronounced symptoms such as respiratory distress and dyspnea (30). This compares to our cohort, where all the patients presented with respiratory symptoms. Conversely, adults are typically asymptomatic when the foramen is large, allowing for normal intra-atrial pressure without significant obstruction (12). During physical examination, the condition may manifest as a diastolic murmur distinct from mitral stenosis by the absence of an opening snap and a distinct loud S1 with a pronounced second heart sound (P2) if pulmonary hypertension develops (31). Our findings demonstrate a nearly equal number of CTS patients in both sexes. These results are consistent with the literature (28). A systematic review by Ullah et al. compiled 235 studies, and the pooled results showed that 54% of the cases were male and 46% were female (32). This consistent observation across different regions suggests that CTS does not have a significant sex predilection, potentially indicating that genetic and congenital factors related to the development of this anomaly are equally likely to occur in both males and females. The patients in our cohort had a median hospital stay of 7 days.

Our results indicate that 100% of our patients had CTS, which aligns with the literature suggesting a higher CTS when compared to Cor Triatriatum Dexter. Specifically, approximately 83% of patients with Cor Triatriatum have CTS, while 17% have Cor Triatriatum Dexter (32). The exclusive finding of CTS in our study may be attributed to the small sample size of 7 patients, which could limit the representation of Cor Triatriatum Dexter cases and highlight the need for larger-scale studies to capture the full spectrum of Cor Triatriatum presentations. Additionally, studies show that the majority of patients with Cor Triatriatum have associated cardiac lesions (12). Our results show that two patients had septal defects, which is notably lower than the 50%-80% range of patients reported in the literature (33). For instance, in a tertiary care center in Toronto, 53% of CTS patients had atrial septal defects and 10% had ventricular septal defects (27). The discrepancy with our findings can be attributed to our sample size, which might not have been sufficient to represent the full range of associated anomalies. Variations in diagnostic criteria or population characteristics may also contribute to this difference, with genetic and environmental factors potentially influencing the clinical presentation of CTS.

In our cohort, pulmonary edema was observed on chest x-ray when performed in 5 patients of our patients. This is expected due to left-sided blood flow obstruction causing an elevated pulmonary venous pressure, eventually leading to capillary leakage and pulmonary edema (34). This finding further indicates the necessity of prompt surgical intervention in dyspneic CTS patients to alleviate the obstruction. Interestingly, 2 patients had no chest x-ray abnormal findings despite having CTS. This can be explained by the variability of CTS and its symptoms, as per Loeffler's classification. Pulmonary hypertension and edema are common CTS findings defined in the literature, with a case series by Ozyuksel et al. reporting it in 8 out of a 15 patient cohort (21, 27, 35–37). Furthermore, Humpl et al. (27) compared the occurrence of pulmonary edema in 3 cohorts: patients who died before intervention, patients who underwent surgical repair, and patients who did not undergo surgical intervention. Pulmonary edema incidence was 55%, 28%, and 0%, respectively, for the 3 groups defined (27). This corroborates the idea that pulmonary edema is a major indicator of the severity of CTS since it was mostly present in the deceased cohort, lesser found in the group requiring surgery, and absent in those not requiring surgery (27). However, it is important to note that normal radiographic findings do not exclude CTS, as shown in the case of pulmonary edema in less severe CTS patients.

In our study, 6 out of 7 (85.7%) of patients with CTS underwent surgical intervention, which is comparable to some findings reported in the literature. Several studies showed that approximately 70% of patients with CTS underwent surgery (27, 32). In a study conducted by the Mayo Clinic, a reported 47% of patients underwent resection of the CTS membrane (38). The variation in surgical rates across different centers may be influenced by differences in the severity of symptoms and institutional practices. The lower surgical rates observed in the Mayo Clinic study may indicate a more conservative management approach or differences in the patient population or severity of presentation (38). In the operated patients, surgery was initiated by median sternotomy and cardiopulmonary bypass, followed by cardioplegic arrest and then resection of the obstructing membrane from an opening in the right atrium. It was finalized by the closure of the atrial septum. Redirection of pulmonary veins in 2 out of the 6 patients undergoing surgery was required as both patients presented with PAPVR. Misplacement and subsequent redirection of pulmonary veins are commonly encountered in the context of CTS repair (39–41). In our cohort, the integrity of the mitral valves was tested, and then regurgitant valves were repaired via commissuroplasty using 5/0 and 6/0 Prolene with pledgets. In a case study of a severe mitral regurgitation co-occurring with CTS, it was reported that this combination is extremely rare and was repaired via annuloplasty (42). The different approaches employed to address the mitral regurgitation are explained by the fact that our patient's mitral valve had a lesion at the posterior commissure, while annuloplasty was used for a “functional regurgitation” due to an annular dilation caused by CTS (42).

A step-by-step protocol for the procedures done in our center is as follows: the procedure is performed under cardiopulmonary bypass and aortic cross clamping. Next, the right atrium is opened and the left atrium is best entered through the interatrial septum, especially if the location of the pulmonary veins and the cor membrane were not accurately identified preoperatively. The anatomy is then meticulously examined, and the position of each pulmonary vein is precisely depicted. Any membrane dividing the left atrium should be excised totally to provide an unobstructed flow between all four pulmonary veins and the mitral valve. The interatrial septal opening is repaired at the end of the procedure. In neonates, a 3–4 mm foramen ovale may be kept open in anticipation of postoperative pulmonary hypertension, especially in cases with obstruction. More details can be seen in Table 5 highlighting individual surgical notes.

**Table 5:** Individual patient surgical notes.

Patients	Patients with Cor Triatriatum (*N* = 7)
Patient 1	• Initiation of cardiopulmonary bypass between the aorta and both vena cavae • Surgical repair performed on the second day of admission • Enlargement of the patent foramen ovale • Resection of the membrane within the left atrium • Relief of the pulmonary vein obstruction
Patient 2	• Initiation of cardiopulmonary bypass between the aorta and both vena cavae and aortic cross-clamping performed • Opening of the right atrium and atrial septum • Identification of an oblique membrane in the left atrium with a small (10 mm) opening • Excision of most of the membrane in the left atrium • Closure of the atrium and atrial septum • Placement of one chest tube in the mediastinum and one in each pleura and closure of the sternum
Patient 3	• Initiation of cardiopulmonary bypass between the aorta and both vena cavae and aortic cross-clamping performed • Opening of the right atrium and atrial septum followed by Identification of an oblique membrane in the left atrium, perpendicular to the septum • Excision of most of the membrane in the left atrium and visualization of all pulmonary veins in the left atrium • Closure of the atrial septum and right atrium and finally the sternum and subcutaneous tissue
Patient 4	Surgery not performed
Patient 5	• Initiation of cardiopulmonary bypass through cannulation of the ascending aorta and both vena cavae and aortic cross-clamping with antegrade blood cardioplegia was performed • Right atriotomy followed by resection of the interatrial septum • Resection of the left atrial membrane • Mitral valve tested for regurgitation, which was negative • Closure of the atrial septal defect using an autologous pericardial patch • Placement of one chest tube in the mediastinum and one in the right pleura • Closure of the sternum, subcutaneous tissue, and skin
Patient 6	• Initiation of cardiopulmonary bypass through cannulation of the ascending aorta and both vena cavae and aortic cross-clamping with antegrade blood cardioplegia were performed • Right and left atriotomy were carried out • Resection of the restrictive intra-atrial membrane with two small orifices in the left atrium • Redirection of the right superior and middle pulmonary veins to the left atrium using an autologous pericardial patch • Mitral valve commissuroplasty using 5/0 Prolene with pledgets • Identification of previously present severe regurgitation, especially at the posterior commissure, likely due to a jet lesion from the membrane's orifice • Re-initiation of bypass to perform mitral valve repair, with two stitches using 6/0 Prolene at the posterior commissure • Placement of chest drainage and closure of the sternum, subcutaneous tissue, and skin
Patient 7	• Median sternotomy was performed • Initiation of cardiopulmonary bypass between the aorta and both vena cavae and aortic cross-clamping with antegrade blood cardioplegia were performed • Opening of the right atria and the atrial septum • Identification and opening of an oblique membrane in the left atrium at the 3:00 position, away from the mitral valve • Closure of the atrial septum and right atrium and the sternum, subcutaneous tissue, and skin

Furthermore, our findings indicate that 2 out of 7 (28.6%) patients with CTS had PAPVR. Our study shows no cases of total anomalous pulmonary venous return.

Finally, mortality rates for CTS are also controversial, with some studies reporting rates as high as 23%, while others showing significantly lower rates around 4% (27, 38). The variations in mortality rates between different centers may be due to differences in the presence and severity of associated cardiac anomalies rather than CTS itself. Centers reporting higher mortality rates may often see cases with complex congenital heart defects that significantly impact patient outcomes (27). In contrast, our cohort had no reported mortalities, highlighting potential differences in case characteristics or care approaches.

Our study has several limitations. First, its small sample size (*n* = 7 patients) may not adequately represent the broader population of patients with CTS, which could affect the generalizability of our findings. Additionally, being a retrospective review, the study may be subject to incomplete or biased data, limiting the accuracy of our conclusions. As the study was conducted at a tertiary care center, there may be referral bias since more complex or severe cases are likely to be overrepresented compared to the general population.

## 5 Conclusion

Cor Triatriatum remains a rare yet significant congenital heart anomaly with special challenges in diagnosis and management. This retrospective review of 7 pediatric patients provides valuable insights into the clinical presentation, diagnostic techniques, and therapeutic outcomes. The management of Cor Triatriatum continues to require a tailored approach, considering the anatomical diversity and associated cardiac anomalies. Moreover, the unique classification systems, such as the Loeffler classification, have been instrumental in enhancing our understanding of the anatomical variations within Cor Triatriatum. The findings from this study emphasize the importance of early and accurate diagnosis, timely surgical intervention, and a personalized case-by-case approach.

## Data Availability

The original contributions presented in the study are included in the article/Supplementary Material, further inquiries can be directed to the corresponding author.
